# Artemisinin-based combination therapy for uncomplicated *Plasmodium falciparum *malaria in Colombia

**DOI:** 10.1186/1475-2875-6-25

**Published:** 2007-02-28

**Authors:** Lyda Osorio, Iveth Gonzalez, Piero Olliaro, Walter RJ Taylor

**Affiliations:** 1 Internacional Centre for Medical Research and Training (CIDEIM) Avenida 1N #3 03, Cali, Colombia; 2 UNICEF/UNDP/World Bank/WHO Special Programme for Research and Training in Tropical Diseases (TDR) World Health Organization, 1211 Geneva 27, Switzerland

## Abstract

**Background:**

Artemisinin-based combination therapy (ACT) is being widely promoted as a strategy to counteract the increase in *Plasmodium falciparum *antimalarial drug resistance.

**Methods:**

A randomized, double-blind, placebo-controlled, clinical trial of the efficacy, effect on gametocytes and safety of the addition of artesunate/placebo (4 mg/kg/day × 3 d) to amodiaquine (10 mg/kg/day × 3 d) was conducted in Choco department, a low intensity transmission area in northwest Colombia.

**Results:**

From 2,137 screened subjects, 85 entered the study: 43 in the amodiaquine plus placebo and 42 in the amodiaquine plus artesunate groups. Potentially eligible cases failed to qualify mostly because they were not available for follow-up visits (73%). Based on a per protocol analysis, the therapeutic response to both treatments was high: amodiaquine/placebo 35/36, 97.2% (95% CI 85.5–99.9), and amodiaquine/artesunate 32/32, 100% (89.1–100) after PCR genotyping. The Kaplan-Meier survival estimates based on all eligible patients enrolled (amodiaquine/placebo: n = 42; amodiaquine/artesunate: n = 41) were similar in the two study groups (P = 0.3). The addition of artesunate significantly decreased gametocyte carriage on Day 4 (OR = 0.1 95% CI 0.02–0.6), Day 7 (OR = 0.2 95%CI 0.04–0.9), Day 14 (OR = 0.09 95% CI 0–0.8), and Day 21 (OR95%CI 0–0.9). Most subjects in both groups (81% in amodiaquine/placebo and 75.6% in amodiaquine/artesunate) reported at least one drug related adverse event. Symptoms were generally mild and self-limiting and there was no serious adverse event. Two patients on amodiaquine/artesunate voluntarily withdrew from study because they could not tolerate the medication.

**Conclusion:**

Both drug regimens were effective in this area of Colombia. The addition of artesunate reduced gametocyte carriage and did not adversely affect tolerability. In this set of patients, the rate of adverse events was higher than in other studies. Patients' follow-up is problematic in areas with dispersed population and affects the conduct of clinical studies and monitoring of treatment effects. The results are discussed in the light of concurrent increase resistance to amodiaquine in other endemic areas in Colombia and the factors that may influence a change in the national antimalarial drug policy.

## Background

Since mid 2006, Colombia is the only country in South America that has not yet introduced artemisinin-based combination therapy (ACT) into its national malaria drug policy for uncomplicated *Plasmodium falciparum *malaria. Colombia accounted for 13.2% (116,872) of the 886,102 malaria cases reported in the Americas in 2004; of these, 44,437 (10.2%) were due to *P. falciparum *[1]. The Colombian Ministry of Social Protection (formerly the Ministry of Health) currently recommends amodiaquine (AQ) at a dose of 25 mg/Kg over 48 h plus a single dose of sulfadoxine/pyrimethamine (SP) and primaquine (PQ, as a gametocytocidal drug) for the treatment for uncomplicated falciparum malaria [2]. Malaria treatment is provided free-of-charge to all microscopically-confirmed cases.

Combination therapy for *P. falciparum *malaria is not new in Colombia. It was first used in the early 1980's when the combination of chloroquine (CQ) plus SP was recommended for all areas where CQ resistance had not been reported and AQ plus SP for areas with known CQ resistance. The combination of AQ plus SP was adopted for the entire country in 1999, after reports of widespread CQ resistance. The most recent clinical studies show that *P. falciparum *therapeutic failure to CQ ranges from 67% to 97% in Antioquia (in the north), and from 44% to 70% in the Pacific Coast region [3,4].

The efficacy of the AQ plus SP combination therapy assessed after 21 days of follow-up is high in parts of Colombia: 2 therapeutic failures out of 90 cases in Antioquia (Uraba and Bajo Cauca regions) [4], no failure in 49 cases in Nariño (city of Tumaco in the south-west). In the Amazon region of Colombia, the efficacy of this combination has not been assessed. However, a high level (87.5%) of therapeutic failure to SP monotherapy has been reported in one endemic area bordering Brazil, where widespread SP resistance is known to occur [5]. Gathering data on the efficacy of antimalarial drugs in other areas of the Colombian Amazon is limited by the availability of suitable sites to conduct clinical studies with extended follow up.

ACTs are currently recommended for the treatment of uncomplicated falciparum malaria [6]. Artemisinin derivatives are potent, rapidly acting antimalarials, which can reduce gametocyte carriage and patient infectivity; the sustained use of artesunate mefloquine reduced falciparum malaria transmission and progression of drug resistance in western Thailand [7,8]. A meta-analysis of a multi-country study showed that the addition of artesunate (AS) increased the efficacy of monotherapies without adversely affecting tolerability, but that absolute cure rates depended on the background resistance to the companion drug [9]. Colombia was one of the participating countries, but slow recruitment prevented the results of this Colombian study from being published at the same time. Here the results of a randomized, double blind clinical trial of the therapeutic efficacy, effects on gametocytes and safety of the addition of AS to AQ are presented and the implications for treatment policy decision in Colombia discussed.

## Methods

### Study site

The study was conducted in the town of Quibdo, the capital of Choco department in the northwest of Colombia (05°42'N 76°40'W). Malaria is reported throughout the year with annual parasite incidence rates ranging from 4.02 per 1,000 inhabitants in 1994 to 157.8 per 1,000 inhabitants in 1998. In the year 2000, a total of 1,206 malaria cases were reported in Quibdo municipality of which 757 (63%) were due to *P. falciparum*. Subjects were recruited at the San Vicente Health post and Ismael Roldan hospital. These two sites diagnose more than 70% of all malaria cases in this town. Health personnel in other health posts were asked to refer potentially eligible subjects who were interested in the study. The study was carried out over two periods due to restrictions with study drug availability: from April 27^th^, 2000 to November 30^th^, 2001 and from November 11^th^, 2002 to February 27^th^, 2004.

### Entry criteria

Subjects with a positive thick smear were included if they fulfilled all the following inclusion criteria: 1) pure *P. falciparum *infection, 2) parasitaemia >500 and <50,000 asexual parasites/μL (subsequently the lower limit was modified to >250 asexual parasites/μL), 3) age between 1 and 65 years old, and 4) availability to return for follow-up. Subjects were excluded if they have any of the following: 1) were pregnant, 2) had a history of allergy to the study drugs, 3) had taken complete treatment with an antimalarial drug in the previous 72 hours or sulphas, clindamycin or tetracycline in the previous week, 4) had a medical history of untreated hypertension or chronic heart, kidney or liver disease, and 5) presented any danger signs of severe malaria. [10] All subjects or their parents (in the case of children under 18 years of age) were requested to give written consent before entering the study. This study was approved by the Ethical Committees of CIDEIM, in Colombia, and of the WHO.

### Laboratory procedures

At enrollment, 10 mL (7 mL in children ≤12 years old) of venous blood was drawn to confirm the diagnosis of falciparum malaria with Giemsa stained thick and thin smears, stored blood on filter paper for further parasite genotyping, and to do a complete haemogram (manual), ALT (Chemroy Canada Inc.), total bilirubin (Chemroy Canada Inc.), and creatinine (Sera-Pak, Bayer). The smears and filter paper blood samples were also collected from finger pricks on Days 1, 2, 3, 4, 7, 14, 21 and 28; biochemistry was repeated on Days 7 and 28. The laboratory analyses were done at the Hospital Ismael Roldan in Quibdo. The smears were read by a laboratory technicians experienced in malaria diagnosis. Parasitaemia was calculated based on the number of asexual forms seen in 200 leucocytes and then multiplied by 40.

To distinguish between a recrudescent and a new infection during follow-up, parasites identified from Days 14 to 28 were compared to those causing the initial episode using PCR amplification of *msp1, msp2 and glurp P. falciparum *genes from blood samples on filter paper according to Snounou and Beck [11].

### Treatment allocation and administration

All study subjects received AQ (Camoquin^® ^Sanofi, France) 30 mg/kg (10 mg/kg/day or a maximum of 1,800 mg total) for three days and were randomly allocated, using individual precoded opaque sealed envelopes, to AS 4 mg/kg/day (maximum 300 mg/day) or placebo for three days. The coded envelopes and AS/placebo tablets were prepared by Creapharm (France) and provided by Sanofi (France). AQ and AS/placebo were administered together with water and biscuits. A study nurse gave, observed, and recorded all treatments. Treatment was repeated if vomiting occurred within 30 minutes of the administered dose. Subjects who failed treatment or had persistent drug induced vomiting received quinine 10 mg/kg/day, three times a day for three days plus clindamycin at 5 mg/kg four times a day over five days.

### Assessment of treatment response and effect on gametocytes

Therapeutic response was classified as Early Treatment Failure (ETF), defined by any one of the following: 1) development of danger signs or severe malaria on Days 1, 2, or 3, in the presence of parasitaemia; 2) parasite density on Day 2 higher than day 0 count; and 3) Parasite density on Day 3 ≥25% of count on Day 0. Late Treatment Failure (LTF), defined by any one of the following: 1) Development of danger signs or severe malaria after Day 3 in the presence of parasitaemia; 2) Unscheduled return of the patient because of clinical deterioration in the presence of parasitaemia; 3) Presence of parasitaemia on any scheduled follow-up day on or after day 7. Adequate Clinical Response (ACR): did not meet criteria for early or late treatment failure and clearance of parasitaemia after Day 3 through Day 28 [12],

To assess the effect of treatment on gametocytes, the number of gametocytes per 1,000 leucocytes was determined in Giemsa stained thick smears together with parasitaemia at enrollment and every visit.

### Assessment of adverse effects

A physician or a nurse recorded any clinical events occurring in the study subjects during follow-up and asked systematically for the presence of anorexia, nausea vomiting, abdominal pain, diarrhoea, headache, dizziness, and any other symptoms. The laboratory evaluations at enrollment and on Days 7 and 28 were used to identify anaemia (defined as haemoglobin <12 g/dL), leucopaenia (WBC count <5,000/mm^3^), neutropaenia (total neutrophils<2,000/mm^3^), severe neutropaenia (<1,000/mm^3^), liver toxicity (ALT> 48 U/L and/or a total bilirubin >1 mg/dL), and renal toxicity (creatinine >1 mg/dL).

The results of the clinical and laboratory assessments were considered as adverse events if they were new or increased in severity with respect to the baseline data according to CTC criteria of severity [13].

Adverse events were classified as probably associated with the study treatment if: the adverse event followed a chronological pattern with the treatment, and/or had been previously described for the study treatment, and could not be explained by other factors including the clinical condition of the subject, other diseases or medications.

### Sample size calculation

The sample size of 360 subjects (180 per study group) was calculated based on an estimated 90% efficacy for AQ, 98% for AQ plus AS, with a two-sided 5% alpha error, 80% statistical power, and 12% estimated loss of subjects to follow-up.

### Data management and statistical analysis

Data were double entered and validated using Epinfo 6.04 d (Center for Disease Control and Prevention, USA). Statistical analysis was done using Epinfo 6.04 d and Stata 8.0 (Stata Corporation, 2003). Survival analysis using the Kaplan Meier method and Log Rank test was carried out to compare the therapeutic responses in the two study groups on all patients enrolled except protocol violations. The therapeutic response at Day 28 of follow-up with its corresponding 95% confidence intervals was calculated on subjects who adhered to the protocol for each study group ("per protocol analysis"). For adverse event analyses only those events that were classified as probably drug related are reported.

Contingency tables and the chi-squared test were used to assess differences in the proportion of subjects with gametocytes at each follow-up day between treatment groups. An additional analysis was conducted only in subjects who were gametocyte negative at enrolment. A P-value <5% was considered as statistically significant.

## Results

**Figure 1:**
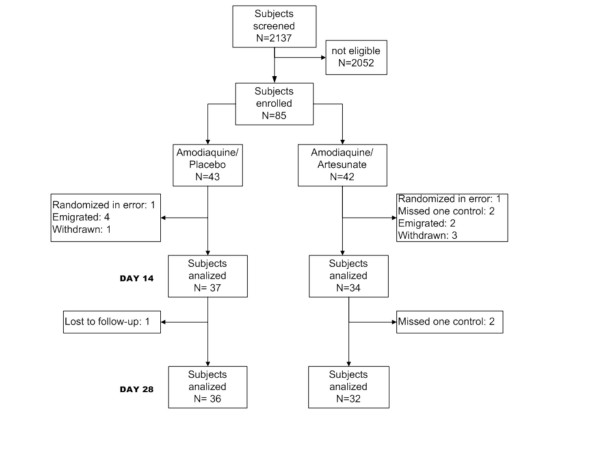
Study profile.

A total of 2,137 subjects were screened in the two study periods, from whom 1,933 (90.4%) were not eligible to enter the study (Figure 1). The majority, 1,176/1,933 (61%) of subjects who were not eligible did not fulfill one entry criterion and 39% (n = 757) did not meet two or more entry criteria. Study ineligibility in the 1176 subjects who did not meet one entry criterion was due to (i) inability to return for the 28 days of follow up (n = 861, 73.2%), (ii) low parasitaemia (n = 210, 17.9%), (iii) danger signs of severe malaria (n = 15, 1.3%), (iv) pregnancy (n = 13, 1.1%), (v) age out of range (n = 9, 0.8%), (vi) chronic disease (n = 6, 0.5%), (vii) previous complete treatment with an antimalarial drug (n = 2, 0.2), and (viii) other reasons (n = 60, 5.1%). Of the 204 subjects who were eligible to enter the study, 119 (58.3%) did not accept to participate and 85 did.

Of the total 85 subjects who entered the study, 43 were randomized to AQ plus placebo and 42 to AQ plus AS; of these, 71 were on the study on Day 14 and 69 on Day 28 (Figure 1). Two patients (one per arm) were excluded from the efficacy and gametocyte analyses because they were wrongly diagnosed. The subjects in the two groups were comparable in their sociodemographic, clinical and laboratory baseline characteristics. However, there were more subjects with gametocytes at enrolment in the AQ plus AS group (Table 1).

**Table 1:** Characteristics of study subjects at enrollment

**Characteristic**		**Amodiaquine/Placebo**	**Artesunate/Amodiaquine**
		
		N = 37 (%)	N = 34 (%)
Age (years old)	Median	19	18.5
	Range	5 to 62	3 to 58
Gender	Male	24 (64.8)	27 (79.4)
	Female	13 (35.2)	7 (20.6)
Weight (kg)	Median	55	57
	Range	19 to 99	12.5 to 80
Temperature (°C)	Median	36.6	36.6
	Range	35.9 to 39.0	36 to 40.2
Parasitaemia (asexual forms/μL)	Geometric mean	3420	4416
	Range	400 to 34,594	480 to 34,594
Presence of gametocytes	Yes	4 (10.8)	8 (23.5)
	No	33 (89.2)	26 (76.5)
Haemoglobin (g/dL)	Median	11,6	12
	Range	8.3 to 15.3	8.0 to 16.3
WBC/mm^3^	Median	5900	5200
	Range	2200 to 12800	2000 to 15800
Neutrophils/mm^3^	Median	3264	2900
	Range	1056 to 7134	1210 to 10428
Total bilirubin (mg/dL)	Median	1	1.3
	Range	0.2 to 5.3	0.1 to 5.2
ALT (U/L)	Median	24	24.2
	Range	2 to 98	5 to 107
Creatinine (mg/dL)	Median	0.9	1
	Range	0.4 to 1.9	0.4 to 1.4

Only two treatment failures were observed, one in each group. Both therapeutic failures were classified as LTF as they occurred on Day 14 (AQ plus placebo group) and Day 28 (AQ plus AS group). PCR genotyping showed identical *msp1 *and *msp2 *amplifications but differences in the fragment length of *glurp *in the samples obtained from Day 0 and Day of failure in the AQ plus AS therapeutic failure and hence the case was reclassified as a reinfection. By contrast, the samples from Day 0 and the Day of failure in the AQ plus placebo therapeutic failure were identical and the case was considered as a true recrudescence. The PCR corrected therapeutic responses for patients evaluable at the Day 28 final visit were 35/36 or 97.2% (95%CI 85.5 – 99.9) for AQ plus placebo, and 32/32 or 100% (89.1 – 100) for AQ plus AS. Excluding the two protocol violations, 42 (AQ plus placebo) and 41 (AQ plus AS) were included in the Kaplan Meier analyses; five and ten subjects were censored during follow-up, respectively. The Kaplan-Meier survival estimates were similar in the two study groups for both crude (P = 0.9) and PCR-corrected (P = 0.3) results.

**Figure 2:**
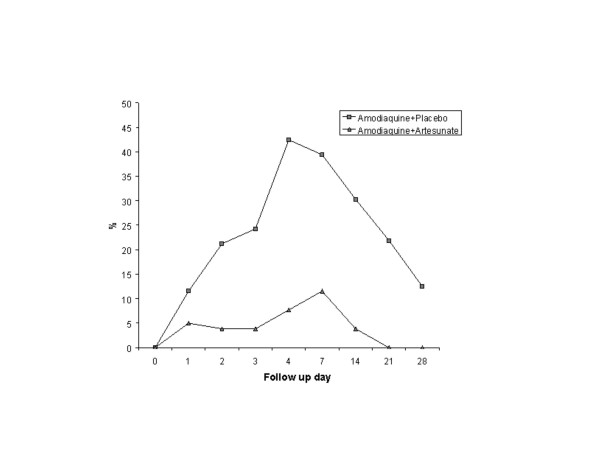
**Proportion of gametocyte carriers during follow up in each study group**. Differences were statistically significant on Days 4 (P = 0.003), 7 (P = 0.01), 14 (P = 0.01), and 21 (P = 0.01).

Just under half of the AQ plus placebo group who did not have gametocytes at enrollment had patent gametocytes at some point during the 28 days of follow up (13/28 46.4%), compared to six of 22 (27.3%) of the AQ plus AS group (P = 0.03). Gametocyte carriage peaked on Day 4 (14/33, 42.4%) in the AQ plus placebo group and on Day 7 (3/26, 11.5%) in the AQ plus AS-treated group. No gametocytes were observed in the AQ plus AS group on Day 21 and after. The differences in gametocyte carriage between groups was statistically significant on Day 4 (OR = 0.1 95%CI 0.02–0.6), Day 7 (OR = 0.2 95%CI 0.04–0.9), Day 14 (OR = 0.09 95%CI 0–0.8), and Day 21 (OR 95% CI 0–0.9) (Figure 2). When the analysis was conducted in all subjects regardless of the presence of gametocytes at enrollment, there was a statistically significant difference in gametocyte carriage on Days 7 and 14: OR = 0.28 95%CI 0.08–1.02 for both days.

Adverse events were frequently reported in both study groups; 34/42 (81%) and 31/41 (75.6%) subjects had at least one adverse event considered to be probably associated with the antimalarial treatment in the AQ plus placebo and AQ plus AS group, respectively. Most events were mild and self limiting or required short term symptomatic treatment. However, two subjects in the AQ plus AS group voluntarily withdrew from the study on Day 2, one because of epigastric pain and the other because of dizziness. There were no serious adverse events. Laboratory adverse events were also common but similar in the two arms (Table 2). They were leucopaenia, neutropaenia, anaemia and an increase in creatinine and ALT. By Day 28, total white blood cell counts ranged from 2,400 to 11,000/mm^3 ^and leucopaenia was still present in 15/36 (42%) and 10/36 (27.8%) subjects in the AQ plus placebo and AQ plus AS group, respectively. The neutrophil counts by Day 28 ranged from 417 to 8,140/mm^3^. Neutropaenia (< 2,000/mm^3^) was observed in 10/36 (27.8%) and 8/36 (22.2%) subjects in the placebo and AS groups, respectively. Of these, six subjects (2 AQ and four in the AQ plus AS groups) had total neutrophil counts <1,000/mm^3 ^but without apparent clinical ill effects. The subject with a neutrophil count of 417/mm^3 ^was already neutropenic at baseline (1,056/mm^3^). There was a mild increase in reticulocytes from Day 0 to Day 28 in both study groups: from median (range) 0.6% (0.0–1.5) to 0.7% (0.0–1.6) and from 0.5% (0.1–2.4) to 0.6% (0.0–1.4) in the placebo and AS-treated groups, respectively. The mean change in haemoglobin between Day 0 and Day 28 was 0.5 g/dL (range: -4.4, 3.7) for the AQ plus placebo group and 0.1 g/dL (range: -3.4, 3.3) for the AQ plus AS group. At Day 28, creatinine ranged from 0.4 to 2.0 mg/dL and ALT from 2.0 to 105 U/L. Increased values of ALT (>48 U/L) were observed in 3/72 (4.1%) subjects (one in AQ plus placebo and two in AQ plus AS groups). A shift from normal laboratory results at baseline to abnormal at Day 28 was seen in 13/43 (34.9%) of subjects with respect to total leucocyte counts (5,000/mm^3^), 15/64 (23.4%) for total neutrophil counts (2,000/mm^3^), 10/38 (26.3%) for haemoglobin (12 g/dL), 4/33 (12.1%) for total bilirubin (1 mg/dL), 3/69 (4.3%) for creatinine (1.4 mg/dL) and 1/65 (1.5%) for ALT (48 U/L). All patients were clinically well.

**Table 2:** Clinical and laboratory adverse events probably associated with the antimalarial treatment by study group that were recorded at least once during follow up.

**Adverse event**^1^	**Amodiaquine/Placebo**		**Amodiaquine/Artesunate**	
	
	N = 34	(%)	N = 31	(%)
Leucopaenia	18	(52,9)	13	(41,9)
Neutropaenia	16	(47,1)	15	(48,4)
Anaemia	10	(29,4)	7	(22,6)
ALT> 48 U/L	6	(17,6)	4	(12,9)
Vomit	5	(14,7)	2	(6,5)
Dizziness	5	(14,7)	6	(19,4)
Abdominal pain	5	(14,7)	4	(12,9)
Bilirubin >1 mg/dL	5	(14,7)	6	(19,4)
Itching	4	(11,8)	4	(12,9)
Headache	3	(8,8)	0	(0)
Diarrhoea	2	(5,9)	2	(6,5)
Anorexia	2	(5,9)	1	(3,2)
Nausea	1	(2,9)	3	(9,7)

^1^Definitions: leucopaenia (WBC<5,000/mm3), neutropaenia (total neutrophils<2,000/mm^3^), anaemia (haemoglobin <12 g/dL). ALT: Alanine Amino Transferase

## Discussion

Artemisinin-based combination therapy (ACT) is being widely promoted as a strategy to counteract increasing resistance of *P. falciparum *to antimalarial drugs. the parasitological and clinical efficacy, effects on gametocytes and safety of the addition of AS to AQ in an area of high CQ-resistant *P. falciparum *malaria in Colombia, one of the countries that has not yet officially adopted ACTs, was evaluated.

In this study, there was a high therapeutic response to 30 mg/kg of AQ monotherapy (97.2%, 95% CI 85.8–99.9) after 28 days. Similarly, the addition of AS showed a high therapeutic efficacy (100%, 95%CI 89.1–100) with only one recurrent parasitaemia on Day 28 that was reclassified as a reinfection based on PCR genotyping. While the 95% confidence intervals of both treatments overlap and the small sample size is insufficient to demonstrate statistical differences in efficacy between treatments, our results suggest that AQ plus AS could be a suitable antimalarial therapy in this area of Colombia. However, the results from Quibdo may not be generalized to other endemic areas in Colombia where high levels of therapeutic failure of 25 mg/kg AQ monotherapy have been reported. In recent studies in three municipalities in the Southern pacific coast (Tumaco, Guapi, and Buenaventura), AQ treatment failures ranged from 33% to 50%, the majority being late treatment failures [14]. The underlying levels of parasite resistance are an important determinant of the policy making process. Although the addition of AS increases the efficacy of AQ alone in areas of Africa where parasitological failures to AQ monotherapy varied from 21.1% to 59%, the therapeutic efficacy of the combination in these areas is still low (from 85.1% to 68.4%) when compared to alternatives such as other combinations with AS and artemether/lumefantrine [9,15].

The effect of AS on gametocyte carriage, consistently seen in other studies, was confirmed here (Figure 2) [9]. This is a desirable characteristic of an antimalarial treatment because this potentially reduces transmission and the spread of resistant parasites [8]. For the same reason, the Colombian Ministry of Health guidelines recommend a single dose of PQ but its effectiveness in reducing gametocyte carriage has not been evaluated. PQ may be associated to the risk of acute intravascular haemolysis when give to G6PD deficient patients [16] One study found 12% prevalence in malaria patients in one endemic area of the pacific coast of Colombia [17]. AS might be a more efficacious, safer and an easier strategy than the addition of PQ to antimalarial regimens for reducing gametocyte carriage among *P. falciparum *infected individuals [18,19]. One small trial has shown that AS reduced gametocytogenesis, whilst 14 days of primaquine enhanced gametocyte clearance [20]. However, no field trial has been conducted to evaluate the relative benefits of an ACT regimen over one containing PQ in reducing malaria incidence.

Adverse events were frequent in both study groups but differences between placebo and AS were not detected (Table 2). A meta-analysis has shown that the addition of AS to other antimalarials do not significantly increase the frequency of adverse events. [9] Leucopaenia and neutropaenia have been previously described with AQ use and related to the toxic effect of its quinoneimine metabolite [21]. Although most of the study subjects did recover, the overall frequencies of leucopaenia (25/72, 34.7%), neutropaenia (18/72, 25%) and severe neutropaenia (6/72, 8.3%) at Day 28 were higher than those reported by Schellenberg et al. (leucopaenia 3.7% and neutropenia 2.5% in AQ-treated Tanzanian children) and by Adjuik et al. ("unremarkable changes in WBC" but similar figures for severe neutropenia, namely, 6% in AQ or AQ plus AS-treated African children) [9,22]. The definition of leucopaenia in the present study (WBC <5,000/mm^3^) was the same as that used by Schellenberg et al, but neutropaenia was defined differently: neutrophils <2,000/mm^3 ^versus <1,500/mm^3 ^[22]. This study also showed a higher proportion of patients with raised ALT >48 U/L than Schellenberg et al: 9.7 vs. 3% [22]. The higher frequencies of laboratory-observed adverse events could be explained by co-morbidities or pharmacogenetic differences in the study groups. Although there were no apparent clinical consequences of the haematological and biochemical changes in AQ-treated subjects, the findings of the present study are a reminder that these AQ-related toxicities occur after only one treatment. The consequences of its repeat use, especially in areas of intense transmission, will need to be monitored once AQ plus AS are deployed. Strategies for implementing an antimalarial pharmacovigilance programme in Africa have been proposed [23]. Colombia has a national pharmacovigilance programme, but this needs to be developed further, especially in malaria-endemic areas.

Besides AS plus AQ, the current WHO malaria treatment guidelines recommend three different ACTs: artemether-lumefantrine (six dose regimen), AS plus mefloquine and AS plus sulphadoxine/pyrimethamine (SP), that are being used in South America [6]. This experience could help the Colombian Ministry of Social Protection to decide which ACTs represent the best options for the country in terms of safety, cost, acceptability, use in pregnancy, treatment of mixed infections (*P. falciparum *and *Plasmodium vivax*), and logistics. Like Peru, Colombia may decide to have two different first-line regimens according to geographical areas, namely, one for the SP sensitive Pacific coast and another for the SP resistant Amazon region, or it could opt for an ACT that would have country-wide efficacy. On the one hand, the advantage of having two first line regimens would be to limit the use of second- and third-line drugs, like quinine combined with an antibiotic or with mefloquine (usually more expensive and with less tolerability) to areas of multidrug resistance. On the other hand, one potential disadvantage would be the need to identify the origin of infection before prescribing treatment, particularly in the major cities, where people from both the Amazon and Pacific Coast are seen and in areas of inter-regional migration.

Before implementing ACTs in Colombia, it is important to highlight that, in spite of reports of therapeutic failures to both AQ and SP as monotherapies, the efficacy of their combined use (AQ plus SP, the current national antimalarial drug policy) has been shown to be 97% or more in the Pacific Coast and northern Colombia [24]. However, the AQ plus SP efficacy studies followed up subjects up to 21 days which means that later treatment failures were not detected. Longer follow up is desirable when assessing the therapeutic efficacy of drugs with long half lives such as AQ and SP, but they are not feasible in the endemic areas in Colombia where the studies have been conducted. Indeed, the main limitation for achieving the required sample size in the present study was the unavailability of subjects to come back for follow up for 28 days. Although the efficacy of AQ plus SP could be over estimated, in comparison to the currently available ACTs, it is cheap and can be used in all trimesters of pregnancy. The potential impact of ACT in controlling falciparum malaria is influencing the thinking of the national malaria control programme towards introducing ACTs. However, to take full advantage of ACTs, the malaria control programme should be able to guarantee the timely provision of quality formulations, proper training for prescribers, appropriate storage of drugs in the hot and humid environments in endemic areas, and design strategies to encourage adherence (e.g. use of co-formulations or blisters). A different therapeutic scheme to treat malaria in pregnancy, especially in the first trimester, in areas where an ACT is deployed may be required because more data are needed on the efficacy and safety of ACTs in this population[19]. A malaria control programme that provides early diagnosis and access to an efficacious antimalarial treatment is essential to decrease malaria transmission in endemic regions.

## Conclusion

AQ plus AS was efficacious in subjects with uncomplicated *P. falciparum *malaria in one endemic area in Colombia and reduced gametocyte carriage compared to AQ alone. However, the relative high frequency of adverse events attributable to AQ found and the reports of AQ resistance in other endemic areas of Colombia suggest that other ACT options should be considered for Colombia.

## Authors' contributions

LO was the principal investigator and designed and managed the study, analysed the data, and wrote the first draft of the manuscript. IG coordinated the study and cowrote the first draft of the manuscript. PO and WRJT designed and coordinated the study and critically reviewed and suggested changes to the manuscript.

## References

[B1] Pan American Health Organization (PAHO) Malaria in the countries and region of the Americas. Time Series Epidemiological Data from 1998 - 2004. http://www.paho.org/English/AD/DPC/CD/mal-2005.htm.

[B2] Ministry of Health of Colombia (1999). Guide for the clinical attention, diagnosis and treatment of malaria.

[B3] Osorio LE, Giraldo LE, Grajales LF, Arriaga AL, Andrade AL, Ruebush II T, Barat L (1999). Assessment of therapeutic response of Plasmodium falciparum to chloroquine and sulfadoxine/pyrimethamine in a low malaria transmission area in Colombia. American Journal of Tropical Medicine and Hygiene.

[B4] Blair S, Lacharme L, Carmona-Fonseca J, Piñeros J, Ríos A, Alvarez T, Alvarez G, Tobón A (2006). Therapeutic efficacy test in malaria falciparum in Antioquia, Colombia. Malaria Journal.

[B5] Osorio L, Pérez L, Gonzalez I (2007). Eficacia de los medicamentos antimaláricos en el Amazonas colombiano. Biomedica.

[B6] WHO (2006). Guidelines for the treatment of malaria.

[B7] Nosten F, van Vugt M, Price R, Luxemburger C, Thway KL, Brockman A, McGready R, ter Kuile F, Looareesuwan S (2000). Effects of artesunate-mefloquine combination on incidence of Plasmodium falciparum malaria and mefloquine resistance in western Thailand: a prospective study. The Lancet.

[B8] Woodrow CJ, Haynes RK, Krishna S (2005). Artemisinins. Postgrad Med J.

[B9] Adjuik M, Babiker A, Garner P, Olliaro P, Taylor W, White N, International Artemisinin Study Group (2004). Artesunate combinations for treatment of malaria: meta-analysis. Lancet.

[B10] WHO (2003). Assessment and monitoring of antimalarial drug efficacy for the treatment of of uncomplicated P. falciparum malaria..

[B11] Snounou G, Beck HP (1998). The use of PCR genotyping in the assessment of recrudescence or reinfection after antimalarial drug treatment. Parasitology Today.

[B12] WHO/PAHO PAHO (1998). Assessment of therapeutic efficacy of medicaments to treat uncomplicated P. falciparum malaria in the Americas..

[B13] Cancer therapy evaluation program (1998). Common toxicity criteria. Version 2.0.

[B14] Gonzalez IJ PJO (2003). Eficacia de amodiaquina y sulfadoxina/pirimetamina en el tratamiento de malaria no complicada por Plasmodium falciparum en Nariño, Colombia, 1999-2002. Biomédica.

[B15] Piola P, Fogg C, Bajunirwe F, Biraro S, Grandesso F, Ruzagira E, Babigumira J, Kigozi I, Kiguli J, Kyomuhendo J, Ferradini L, Taylor W, Checchi F, Guthmann J (2005). Supervised versus unsupervised intake of six-dose artemether-lumefantrine for
treatment of acute, uncomplicated Plasmodium falciparum malaria in Mbarara,
Uganda: a randomised trial.. The Lancet.

[B16] López-Antuñano F (1999). Is primaquine useful and safe as true exo-erythrocytic merontocidal, hypnozoitocidal and gametocidal antimalarial drug?. Salud Pública de México.

[B17] Moyano M, Méndez F (2005). Erythrocyte defects and parasitemia density in patients with Plasmodium falciparum malaria in Buenaventura, Colombia. Revista Panamericana de Salud Publica.

[B18] Bousema JT, Schneider P, Gouagna LC, Drakeley CJ, Tostmann A, Houben R, Githure JI, Ord R, Sutherland CJ, Omar SA, Sauerwein RW (2006). Moderate Effect of Artemisinin-Based Combination Therapy on Transmission of Plasmodium falciparum. Journal of Infectious Diseases.

[B19] Nosten F, McGready R, d´Alessandro U, Bonnel A, Verhoeff F, Menendez C, Mutabingwa T, Brabin B (2006). Antimalarial drugs in pregnancy: A review. Current Drug Safety.

[B20] Pukrittayakamee S, Chotivanich K, Chantra A, Clemens R, Looareesuwan S, White NJ (2004). Activities of artesunate and primaquine against asexual-and sexual-stage parasites in falciparum malaria. Antimicrobial Agents and Chemotherapy.

[B21] Winstanley PA, Coleman JW, Maggs JL, Breckenridge AM, Park BK (1990). The toxicity of amodiaquine and its principal metabolites towards mononuclear leucocytes and granulocyte/monocyte colony forming units. British Journal of Clinical Pharmacology.

[B22] Schellenberg D, Kahigwa E, Drakeley C, Malende A, Wigayi J, Msokame C, Aponte JJ, Tanner M, Mshinda H, Menendez C, Alonso PL (2002). The safety and efficacy of sulfadoxine-pyrimethamine, amodiaquine, and their combination in the treatment of uncomplicated Plasmodium falciparum malaria.. American Journal of Tropical Medicine and Hygiene.

[B23] Talisuna AO, Staedke SG, D' Alessandro U (2006). Pharmacovigilance of antimalarial treatment in Africa: Is it possible?. Malaria Journal.

[B24] Carmona J, Tobón A, Alvarez G, Blair S (2005). El tratamiento amodiaquina-sulfadoxina-pirimetamina tiene eficacia del 98% para la malaria falciparum no complicada (Antioquia, Colombia; 2003). Iatreia.

